# Evaluation of Fructosamine 3-kinase and Glyoxalase 1 activity in normal and breast cancer tissues

**DOI:** 10.37796/2211-8039.1130

**Published:** 2021-09-01

**Authors:** Tooba Yousefi, Abdol Rahim Gholizadeh Pasha, Ghodsieh Kamrani, Ailin Ebrahimzadeh, Ali Zahedian, Karimollah Hajian-Tilaki, Mohammad Aghajani, Durdi Qujeq

**Affiliations:** a Cellular and Molecular Biology Research Center, Health Research Institute, Babol University of Medical Sciences, Babol, Iran; b Department of Clinical Biochemistry, Babol University of Medical Sciences, Babol, Iran; c Student Research Committee, Babol University of Medical Sciences, Babol, Iran; d Department of Surgery, School of Medicine, Babol University of Medical Sciences, Babol, Iran; e Department of Pathology, School of Medicine, Babol University of Medical Sciences, Babol, Iran; f Department of Epidemiology and Biostatistics, School of Medicine, Babol University of Medical Sciences, Babol, Iran; g Shahid Beheshti Hospital, School of Medicine, Babol University of Medical Sciences, Babol, Iran

**Keywords:** Breast Neoplasm, Glyoxalase I, Methylglyoxal, Fructosamine-3-kinase, Advanced Glycation End products, Glycation

## Abstract

**Background:**

Breast cancer is a typical malignancy and the most common in the female and it is the primary reason behind cancer-related deaths of women around the world. The pathological role of the non-enzymatic change of proteins by reducing sugars become frequently shows in different kinds of cancer. Cancer cells generally rely upon aerobic glycolysis as the main source of energy. Impaired glucose metabolism is somewhat responsible for the aggregation of advanced glycation end products (AGEs). Methylglyoxal (MG), a glycolysis byproduct either contributes to the accumulation of AGEs. Enzymatic defense upon AGEs products exists in all mammalian cells.

**Aims:**

The present work intends to look into Glyoxalase1 (GLO1) and fructosamine-3-kinase (FN3K) activity in human breast carcinoma.

**Methods:**

Thirty-three consecutive patients were entered into the study. Samples of breast tumoral tissue and normal matches were drawn from patients after surgery. FN3K and GLO1 enzymatic activity were analyzed using a radiometric and spectrophotometric assay.

**Results:**

The average level of FN3K enzyme was fundamentally lower in cancerous tissues parallel with adjacent noncancerous tissues. We also observed a consistent increase of GLO1 activity in the tumor parallel with pair-matched normal tissue.

**Conclusion:**

The current findings build up a key-role of enzymatic defense to detoxify cytotoxic AGEs and methylglyoxal levels in tumor cells. These discoveries may give another system to the treatment of breast cancer.

## 1. Introduction

Breast cancer is a common malignancy and the most prevalent in the female and it is the main reason for cancer-related deaths of women worldwide [1, 2]. About 1.7 million cases of breast cancer are identified each year, looking at the other point of view, it means in every 18 s one new case recognized [3]. The incidence of breast cancer has been growing and 53% of these cases take place in developing countries [4, 5]. Changes in cellular metabolism are usual events in cancer to produce energy cancer cells intensely count on glycolysis which is necessary for cellular processes even in the presence of oxygen [6, 7]. This process correlated with the ‘Warburg effect’ or aerobic glycolysis is recognized as a sign of cancer cells [8, 9].

Elevated glycolytic rates in parallel with an increase in cancer cell metabolism lead to the gathering of advanced glycation end products (AGEs) which are the fixed outcome of the Non-enzymatic glycation (Maillard reaction) [10, 11]. In the Maillard reaction reducing sugars react automatically with amino residues in proteins, nucleic acids, and lipids to produce AGEs [12]. AGEs can provoke damage to the organism by modifying proteins and changing their metabolism, physical and chemical characteristics [13, 14]. Indeed they can prevent the entry of the proteasomal core. They can elevate protein modification by reducing proteolytic activity and increasing in oxidized and damaged proteins [15, 16]. In addition, AGE-arrangement impacts gene expression and the activity of signal transduction molecules such as growth factors or ion channels. They work as signaling molecules and upgrade oxidative stress and expression of pro-inflammatory cytokines by explicit receptors, for example, RAGE [17, 18].

Fructosamine is one of the strongest precursors for excessive glycation which provokes constant effects on protein structure and function. It expresses in all glycated serum proteins and is correlated with the formation of AGEs and progression of cancer [19, 20].

Fructosamine 3-kinase (FN3K; EC 2.7.1.171) is an enzyme required in the deglycation procedure, novel situation of protein repair [21, 22]. To displace fructosamine deposits from proteins FN3K phosphorylates fructosamine to fructosamine 3-phosphates that are flimsy and decline precipitously into 3-deoxyglucosone and inorganic phosphate [23, 24]. Also, the upgraded progression of metabolites during glycolysis is related to a gathering of side items like reactive carbonyl species (RCS) [17, 25].

Methylglyoxal (MG) is a noteworthy precursor of AGEs and an exceptionally RCS which is begun from intermediates of glycolysis via programmed corruption of triose phosphate, intermediates of glycolysis, protein debasement, and glycation [8, 26]. This a-oxoaldehyde is more responsive than glucose in glycation procedures and skilled to adjust proteins, lipids, and nucleotides, causing cell damage [6]. MGO can change amino acid deposits of proteins to create AGEs [27]. Collecting proof proposes that MG shows antitumor action inside a strong cytotoxic impact in numerous cancer cells [28, 29].

**Fig 1:**
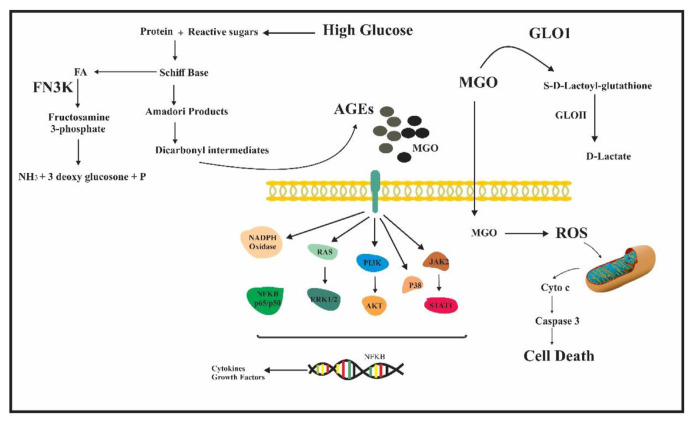
Mechanisms of AGEs Pathogenicity and Deglycation Process. AGEs may exert their effect by the activation of RAGE (receptor for advanced glycation end-products), results in the activation of the key mediators of the proliferation and inflammation such as PI3k/AKT, RAS/MAPK, JAK/ STAT, NF-kB pathways.

Glyoxalase I (GLO1: EC 4.4.1.5) is an anti-glycation defense enzyme which diminishes the centralization of methylglyoxal [30]. The physiological capacity of GLO1 is to eliminate cytotoxic methylglyoxal from cells as non-poisonous D-lactate [31–33]. It has been validated that Glo-1 expression and activity are connected with cancer growth movement [34, 35]. As a result of these unfavorable impacts, cancer cells depend on the outflow of aldehyde barrier proteins, for example, GLO1 to evade extreme aldehyde stress and FN3K to confine AGE-amassing [17, 36](Fig. 1). This paper, to our knowledge, is the primary investigation on FN3K and GLO1 enzyme activity in human breast cancer by uncovering a noteworthy connection between these enzymes and tumor tissue.

## 2. Methods

### 2.1. Clinical tumor samples

Tissue tests were gathered from the Shahid Beheshti hospital associated with the University of Medical Sciences following the moral rules of the Babol University of Medical Science. All the patients gave informed consent to take part in the investigation. Breast tissue tests were taken in the period February 2018 – March 2019 from 33 women (mean age 59.09 ± 9.49 (Mean ± SD) years, 33–79 years extend), who experienced medical procedures for breast cancer. None of the patients got anti-neoplastic therapy before surgery. A pathologist checked the existence of tumor cells in examples. Healthy surrounding breast tissues were taken from a similar breast cancer patient. Examples were gotten inside 1 h after the surgery and immediately positioned into fluid nitrogen at that point put away at − 80C until measured.

### 2.2. Sample preparation

About 0.1 g of tumoral or typical tissue from every patient was defrosted and homogenized at 4°C in 600 μl PBS cushion, pH 7.4, within the sight of 40 μl/ml complete proteases inhibitor mixed drink (Roche, Sigma Aldrich Cat. No: 8340). Samples were centrifuged at 3000 rpm for 20 min at 4°C. Test protein content was resolved utilizing Bradford’s strategy.

### 2.3. FAN3K activity assay

FN3K action was identified utilizing a colorimetric method [37]. Bovine serum albumin (BSA; 10 g/L, Sigma Aldrich Cat. No: 2153) was glycated by incubation with 10% glucose solution at 37 °C. After 72 h of incubation, glycated BSA was dialyzed toward 0.1 mol/Lphosphate-cushioned saline (pH: 7.4) at 4 °Cfor 12 h. The dialyzed arrangement was utilized as a substrate. In the measure, 300 μL of the substrate was incubated with 50 μL of serum, 50 μL NBT and 100 μL of MgCl2 (.1 g, Merk Cat. No:105833)/ATP (13 mg). A first perusing of the fructosamine concentration was taken out. The response depends on the limit of ketamine to reduce Nitro Blue Tetrazolium (NBT, Bio-Shop Cat. No NBT001.250) to formazan in a basic arrangement. After incubating the mixture for 120 min at 25 °C, the fructosamine substance of the response compound was estimated again utilizing a similar strategy. The distinction in fructosamine concentration after some time is a proportion of the FN3K activity (1 U comparing to 1 μmol/min).

### 2.4. GLO1 and FA assay

GLO1 and FA were analyzed by enzyme immunoassay by the Bioassay kit (Human Glyoxalase I Bioassay technology laboratory, Cat. No: E3954Hu/ Human Fructosamine Bioassay technology laboratory, Cat. No: E3232Hu) according to the protocol. The kits are a sandwich enzyme immunoassay for the quantitative measurement of GLO1 protein and FA in human tissue.

### 2.5. Statistical analysis

The Kolmogorov-Smirnov test is utilized to contrast tests and a reference likelihood distribution. The distinctions in FN3K activity and FA, GLO1 levels among normal and tumoral tissue were dissected by the Wilcoxon test. The Mann-Whitney and Kruskal-Wallis were used for comparisons between the two and three groups.

## 3. Result

The clinical features of all patients are shown in Table 1.

### 3.1. Comparison of FAN3K, GLO1, and FA levels between BC tissue and healthy tissue

**Fig 2:**
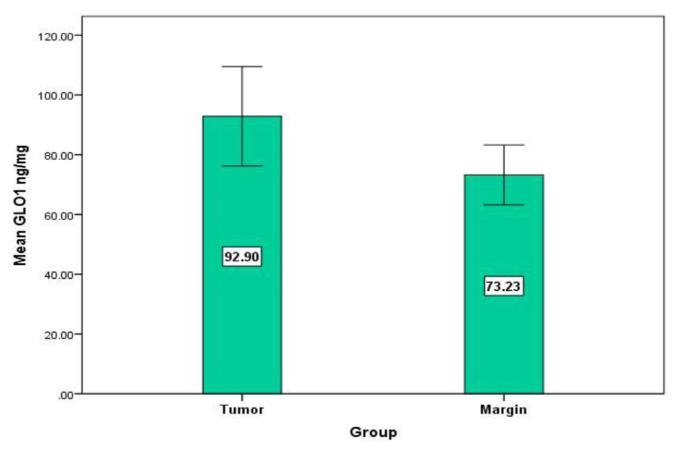
Mean difference of GLO1 level in the margin (73.23 ± 5.01, Mean ± SE) and cancer (92.89 ± 8.31, Mean ± SE). P = 0.001 (Wilcoxon Signed Ranks Test).

**Fig 3:**
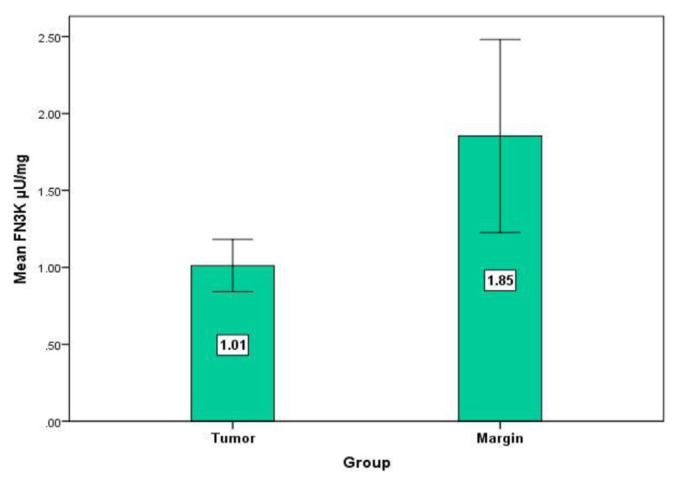
Mean difference of FN3K activity in the margin (1.85 ± 0.31, Mean ± SE) and cancer (1.01 ± 0.08, Mean ± SE). P = 0.01 (Wilcoxon Signed Ranks Test).

**Fig 4:**
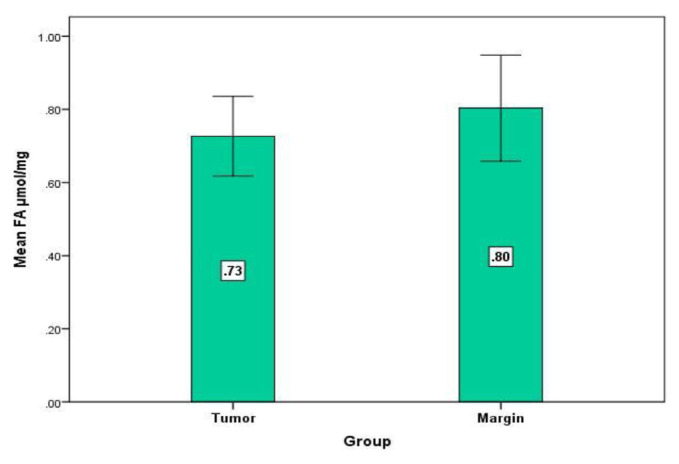
Mean difference of FA level in margin (0.80 ± 0.07, Mean ± SE) and cancer (0.72 ± 0.05, Mean ± SE). P = 0.2 (Wilcoxon Signed Ranks Test).

Our outcomes affirmed a noteworthy increase in GLO1 level in dangerous tissues contrasted and combined non-cancerous tissues (P = 0.001, Wilcoxon Signed Ranks Test) (Fig. 2). Individually, there was a critical decrease in FAN3K movement in the tumor tissue contrasted with controls (P = 0.01, Wilcoxon Signed Ranks Test) (Fig. 3). No difference between cancers versus typical tissue was distinguished in FA levels (P = 0.2, Wilcoxon Signed Ranks Test) (Fig. 4). To recognize the impact of aging we assessed the degrees of FAN3K, GLO1, and FA in patients, the participants of the examination were arranged in two distinctive age groups for example ≤48 years and >48 years. As it is obvious from Table 2, the FAN3K level was fundamentally higher in >48 years BC subjects (P = 0.04, Mann-Whitney Test). Additionally, there was no huge contrast among age and GLO1, FA levels.

Spearman’s correlation coefficient was used to assess the relationship between Glo1, FN3K, and FA in BC tissue. Differences were considered statistically significant at p < 0.05 between Glo1 and FA (Spearman’s rho = 0.70). Correlation between GLO1 and FN3K, FN3K and FA was not significant.

### 3.2. Examination of FAN3K, GLO1, and FA levels and clinico-pathological highlights

To assess the potential relationship between clinical attributes and the mean degrees of FAN3K, GLO1, FA patients were partitioned into subgroups dependent on the clinical stage, evaluation, and TNM data as appeared in Tables 3–5. There was a noteworthy distinction among FA and tumor grade (P = 0.04, Kruskal-Wallis Test) however no huge contrasts in FAN3K and GLO1 levels as indicated by clinical-histopathologic highlights of patients.

### 3.3. The diagnostic value of FN3K, GLO1, and FA for discrimination of breast cancer patients

**Fig 5:**
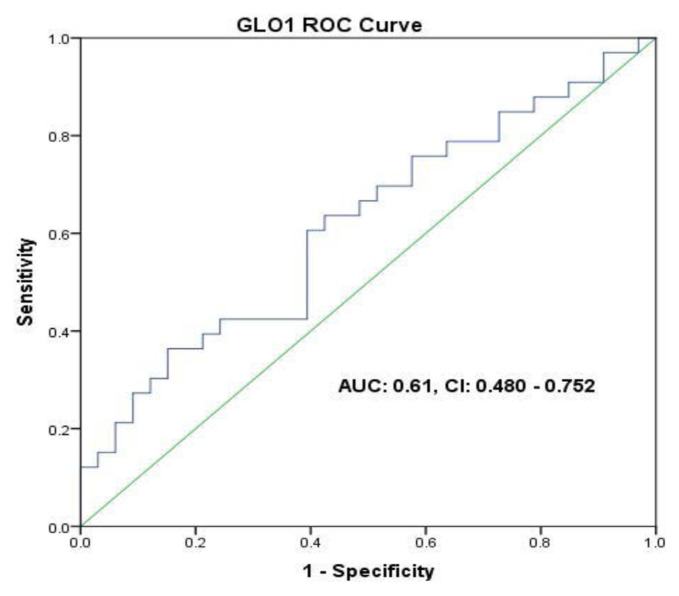
Potential diagnostic accuracy of GLO1 for discrimination between BC patients and the healthy ones.

**Fig 6:**
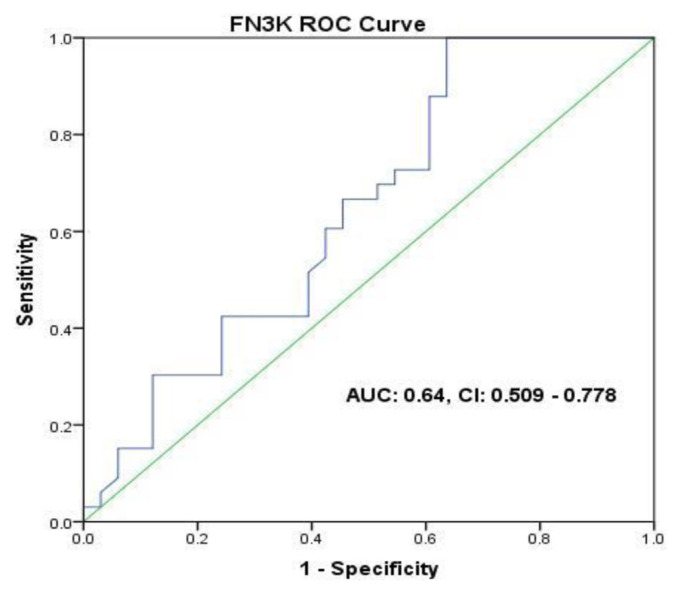
Potential diagnostic accuracy of FN3K for discrimination between BC patients and the healthy ones.

ROC curves and the region under the ROC curve (AUC) were utilized for the discrimination between BC patients and the healthy ones. As indicated by the ROC investigation, the cutoff value of 1.175 FN3K, and 73.97 GLO1 change demonstrated the best analytic accuracy for segregating the two groups: FN3K (AUC = 0.64 (95% CI = 0.509–0.778); sensitivity = 66%; specificity = 55%) GLO1 (AUC = 0.61 (95% CI = 0.480–0.752); sensitivity = 63%; specificity = 58%) (Figs. 5 and 6).

## 4. Discussion

Cancer cells effectively create vitality by improving their pace of glycolysis which builds the collection of advanced glycation products [34]. These heterogeneous macromolecules change the structure and capacity of some extra-and intracellular proteins by non-enzymatic glycation [38]. A low degree of deglycating compounds is the way to created protein glycation in the tumor phenotype. This article is the main examination FN3K enzyme activity in human breast cancer growth, uncovering a critical relationship between enzymatic movement in tumor and pair-coordinated ordinary tissues. Our outcome affirmed there is a critical decrease in FAN3K action in the tumor tissue contrasted with controls. This is in a straight line with discoveries via Caruso et al. in which the mean degree of FN3K quality articulation was very lower in cancer tissue [39]. As opposed to our investigation, Notarnicola et al. show that FN3K has no noteworthy contrasts between typical mucosa and cancer. They proposed the mean degree of FN3K mRNA was very lower in cancer against equivalent typical colorectal mucosa [40]. More recently, an examination performed by Sanghvi et al. announce that glycation can influence the capacity of cell proteins, for example, translation factors, heat shock proteins, compounds in glucose digestion, DNA and RNA restricting proteins, for example, transcription factors, replication and repair proteins, splicing factors, and furthermore histone proteins. This includes histone and DNA alteration in gene expression and DNA repair[41].

The high concentrations of MGO will also induce an increased formation of AGEs. It has been reported that The high levels of MG which are produced due to high glycolytic activity, controlled by increased Glo-1 expression and activity in several tumor cells [42, 43].

Particularly, our results show that the activity levels of GLO1 in breast carcinoma, significantly higher than in pair-matched normal tissues as it was previously reported in several human cancers. Our results are following Michel et al. findings. They detected the high expression of GLO1 in human HCC tissue samples also Targeted GLO1 by EP which significantly reduced proliferation, migration, and colony formation of HCC tissue samples[44].

In another investigation, Guo et al. shown that GLOI restraint or MG treatment can hinder proliferation, invasion, and migration and cause apoptosis in human breast cancer cells. These occasions adjusted by actuation of the MAPK signaling pathway and downregulation of Bcl-2 and MMP-9 which essentially upgraded apoptosis [28].

These events indicate a reaction of tumor cells to high cellular methylglyoxal stress compared with glycolytic adaptations associated with the ‘Warburg effect’ [45].

In different cases, Notarnicola et al. distinguished a significant decline in both GLO1 and FN3K activity in patients with tumors instead of patients with adenomas and the controls [46]. We found no significant difference in FAN3K, GLO1, and FA enzymatic action and levels in a high stage when contrasted and low stage tumors. Patients in clinical stages III had a low degree of GLO1 and FAN3k than patients in clinical stages I–II. Remarkably, our discoveries show that FA fundamentally diminishes with cutting edge tumor grade while No noteworthy contrast was seen somewhere in the range of FAN3K and GLO1 with tumor grade. As opposed to our report, there is a report where GLO1 overexpression was related to high tumor grade [47]. It appears that FAN3K with sensitivity %66 and specificity %55, GLO1 with sensitivity %63, and specificity %58 has sensible symptomatic exactness for segregating BC patients than healthy subjects. Further examination is required to assess the significant role of FN3K and GLO1 on the progression and pathogenesis of BC. Nonetheless, this finding could assist with showing signs of improvement comprehension of non-enzymatic glycation and its relationship to metabolic issues happening in BC.

## 5. Conclusions

Our outcomes affirmed a significant increase in GLO1 levels in cancerous tissues resembled with matched non-carcinogenic tissues. Additionally, there was a critical decrease in FAN3K activity in the tumor tissue contrasted with controls. The findings of this investigation give proof that deglycating enzymes might be associated with expanding the danger of breast cancer and harmful transformation.

## Supplementary Information











## Figures and Tables

**Table 1:** Clinico-histopathologic features of Breast Cancer patient.

	Cases(n = 33)
Age(mean ± SD)	59.09 ± 9.49
Tumor Side	
Left	17
Right	16
Tumor Stage	
Stage 1	9
Stage II	15
Stage III	9
Histological Grade	
G I	4
G II	22
G III	7
Lymph Node	
N 0	13
N I	12
N II	6
N III	2
Tumor Size	
T1	13
T2	17
T3	3
Histological Subtype	
IDC	30
ILC	3

IDC: Invasive ductal carcinoma.

ILC: Invasive lobular carcinoma.s

**Table 2:** Comparison of FN3K, GLO1, FA levels based on age category.

Age	No.	Mean Rank	Mean ± SE (Median)	P-value
FN3K (μU/mg protein)				
>48	21	19.50	1.1 ± 0.11 (1.13)	0.04
≤48	12	12.63	0.80 ± 0.1 (0.77)	
GLO1 (ng/mg protein)				
>48	21	17.52	95.98 ± 11.05 (80.50)	0.67
≤48	12	16.08	87.49 ± 12.65 (77.58)	
FA (μmol/mg protein)				
>48	21	17.90	0.72 ± 0.05 (0.7)	0.47
≤48	12	15.42	0.72 ± 0.12 (0.59)	

**Table 3:** Examination of FN3K (μU/mg protein) activity and clinico-pathological highlights of breast cancer.

Variable	No.	Mean Rank	Mean ± SE (Median)	P-value
Tumor Stage				
I	9	18.50	1.07 ± 0.15 (1.01)	0.30
II	15	18.63	1.07 ± 0.13 (1.13)	
III	9	12.78	0.84 ± 0.15 (0.71)	
Tumor Size				
T1	13	17.42	1.02 ± 0.14 (0.99)	0.91
T2	17	17.06 1 ± 0.1 (0.59)		
T3	3	14.83 1 ± 0.48 (0.97)		
Lymph Nodes				
N0	13	19.77	1.14 ± 0.13 (1.08)	0.56
N1	12	14.25	0.84 ± 0.12 (0.82)	
N2	6	16.75	1.07 ± 0.26 (0.85)	
N3	2	16.25	0.96 ± 0.01 (0.96)	
Histological tumor grade				
1	4	27.13	1.55 ± 0.2 (1.56)	0.07
2	22	15.09	0.92 ± 0.1 (0.78)	
3	7	17.21	0.98 ± 0.12 (0.97)	
Histological subtype				
IDC	30	17.53	1.03 ± 0.08 (0.98)	0.31
ILC	3	11.67	0.78 ± 0.53 (0.42)	
Stage category				
early	24	18.58	1.07 ± 0.09 (1.1)	0.12
advance	9	12.78	0.84 ± 0.15 (0.71)	

**Table 4:** Examination of GLO1 (ng/mg protein) level and clinico-pathological highlights of breast cancer.

Variable	No.	Mean Rank	Mean ± SE (Median)	P-value
Tumor Stage				
I	9	18.11	96.58 ± 16.39 (80.50)	0.55
II	15	18.13	100.53 ± 14.01 (83.55)	
III	9	14.00	76.48 ± 11.15 (74.39)	
Tumor Size				
T1	13	18.31	102.97 ± 17.12 (80.50)	0.81
T2	17	16.06	85.89 ± 8.84 (71.91)	
T3	3	16.67	88.93 ± 25 (78.78)	
Lymph Nodes				
N0	13	16.69	89.95 ± 11.36 (79.87)	0.99
N1	6	17.50	100.89 ± 17.66 (87.16)	
N2	12	16.67	86.51 ± 18.40 (91.88)	
N3	2	17.00	83.22 ± 7.9 (83.22)	
Histological tumor grade				
1	4	25.00	130.94 ± 24.17 (134.97)	0.17
2	22	16.55	91.92 ± 10.90 (80.18)	
3	7	13.86	74.23 ± 8.06 (75.30)	
Histological subtype				
IDC	30	16.47	88.23 ± 7.5 (79.32)	0.31
ILC	3	22.33	139.53 ± 52.19 (109.38)	
Stage category				
early	24	18.13	99.05 ± 10.48 (82.02)	0.27
advance	9	14.00	76.48 ± 11.15 (74.39)	

**Table 5:** Examination of FA (μmol/mg protein) level and clinico-pathological highlights of breast cancer.

Variable	No.	Mean Rank	Mean ± SE (Median)	P-value
Tumor Stage				
I	9	19.83	0.84 ± 0.14 (0.82)	0.17
II	15	18.33	0.73 ± 0.07 (0.71)	
III	9	11.94	0.58 ± 0.05 (0.52)	
Tumor Size				
T1	13	19.73	0.85 ± 0.11 (0.82)	0.42
T2	17	15.26	0.64 ± 0.04 (0.7)	
T3	3	15.00	0.63 ± 0.15 (0.58)	
Lymph Nodes				
N0	13	18.58	0.78 ± 0.1 (0.71)	0.55
N1	12	16.67	0.67 ± 0.07 (0.66)	
N2	6	17.25	0.76 ± 0.13 (0.69)	
N3	2	8.00	0.51 ± 0.05 (0.51)	
Histological tumor grade				
1	4	26.00	0.87 ± 0.01 (0.87)	0.04
2	22	17.27	0.75 ± 0.07 (0.68)	
3	7	11.00	0.55 ± 0.02 (0.55)	
Histological subtype				
IDC	30	15.97	0.7 ± 0.05 (0.63)	0.05
ILC	3	27.33	0.95 ± 0.08 (0.91)	
Stage category				
early	24	18.90	0.77 ± 0.06 (0.75)	0.06
advance	9	11.94	0.58 ± 0.05(0.52)	
