# Supplementary material for: Evaluation of Fructosamine 3-kinase and Glyoxalase 1 activity in normal and breast cancer tissues
Source: Biomedicine (Taipei). 2021 Sep 1;11(3):15–22. doi: 10.37796/2211-8039.1130 (PMC8823491; doi:10.37796/2211-8039.1130)
Supplement: Supplementary file 1 [file bmed-11-03-015-s001.pdf]

ENGLISH EDIT WWW.NativeEnglishEdit.com

EDITORIAL CERTIFICATE LETTER

This document is to certify that the manuscript listed below was edited for proper English language, grammar, punctuation, spelling, and overall style by one of the highly qualified subject-expert native English speaking editors at **NativeEnglishEdit.com**

The substantive content of the article mentioned below remains the full responsibility of the author/authors:

TITLE OF ARTICLE:
EVALUATION OF FRUCTOSAMINE 3-KINASE AND GLYOXALASE 1 ACTIVITY IN NORMAL AND BREAST CANCER TISSUES

AUTHOR(S):
TOOBA YOUSEFI , ABDOL RAHIM GHOLIZADEH PASHA , GHODSIEH KAMRANI , AILIN EBRAHIMZADEH, ALI ZAHEDIAN , KARIMOLLAH HAJIAN-TILAKI , MOHAMMD AGHAJANI , DURDI QJUEQ

REFER CODE:

NEW EE RE-2020-333312292-TOOBA-2267 MEDI

Native English Edit
www.NativeEnglishEdit.com

Documents receiving this certification should be English-ready for publication; however, the author has the ability to accept or reject our suggestions and changes.
This certificate may be verified at:
www.NativeEnglishEdit.com
London
East End Road 27, N 3 3QT
United Kingdom
