# Supplementary material for: Evaluation of Fructosamine 3-kinase and Glyoxalase 1 activity in normal and breast cancer tissues
Source: Biomedicine (Taipei). 2021 Sep 1;11(3):15–22. doi: 10.37796/2211-8039.1130 (PMC8823491; doi:10.37796/2211-8039.1130)
Supplement: Supplementary file 4 [file bmed-11-03-015-s004.docx]

**Original article**

**Evaluation of Fructosamine 3-kinase and Glyoxalase 1 activity in normal and breast cancer tissues**

**Tooba Yousefi^1,2,3^, Abdol Rahim Gholizadeh Pasha^4^**, **Ghodsieh Kamrani^5^,** **Ailin ebrahimzadeh^1,2,3^**, **Ali Zahedian^4^**, **Karimollah hajian-Tilaki^6^**,  **mohammd Aghajani^7^ , Durdi Qjueq ^*1,2,3^**

1. Cellular and Molecular Biology Research Center, Health Research Institute, Babol University of Medical Sciences, Babol, IRAN
2. Department of Clinical Biochemistry, Babol University of Medical Sciences, Babol, IRAN
3. Student Research Committee, Babol University of Medical Sciences, Babol, IRAN
4. Department of Surgery, School of Medicine, Babol University of Medical Sciences, Babol, IRAN
5.Department of Pathology, School of Medicine, Babol University of Medical Sciences, Babol, IRAN
6. Department of Epidemiology and Biostatistics, School of Medicine, Babol University of Medical Sciences, Babol,
IRAN
7. Shahid Beheshti hospital, School of Medicine, Babol University of Medical Sciences, Babol, IRAN

*Corresponding Author: Prof. Durdi Qujeq, PhD
Department of Clinical Biochemistry, Faculty of Medicine, Babol University of Medical Sciences, Ganjafrooze Avenue, Babol, IRAN
Fax: +98-111-2226109, Tel:+98-111-2229591-5, P.O.Box: 47176-47745, Email: d.qujeq@mubabol.ac.ir ,
dqujeq@gmail.com
