# Supplementary material for: Evaluation of Fructosamine 3-kinase and Glyoxalase 1 activity in normal and breast cancer tissues
Source: Biomedicine (Taipei). 2021 Sep 1;11(3):15–22. doi: 10.37796/2211-8039.1130 (PMC8823491; doi:10.37796/2211-8039.1130)
Supplement: Supplementary file 5 [file bmed-11-03-015-s005.docx]

**Original article**

**Evaluation of Fructosamine 3-kinase and Glyoxalase 1 activity in normal and breast cancer tissues**

**Abstract:**

**Background:** Breast cancer is a typical malignancy and the most common in the female and it is the primary reason behind cancer related deaths of women around the world. The neurotic role of the non-enzymatic change of proteins by reducing sugars become frequently shows in different kinds of cancer. Cancer cells generally rely upon aerobic glycolysis as the main source of energy. Damaged glucose metabolism is somewhat responsible for the aggregation of advanced glycation end products (AGEs). Methylglyoxal (MG), a glycolysis byproduct either contributes to the accumulation of AGEs. Enzymatic defense upon AGEs products exists in all mammalian cells. **Aims**: The present work intends to look into Glyoxalase1 and fructosamine-3- kinase (FN3K) activity in human breast carcinoma.

**Methods:** Thirty-three consecutive patients were entered into the study. Samples of breast tumoral tissue and normal matches were drawn from patients after surgery. FN3K and GLO1 enzymatic activity were analyzed using a radiometric and spectrophotometric assay.

**Results:** The average level of FN3K enzyme was fundamentally lower in cancerous tissues parallel with adjacent noncancerous tissues. We also observed a consistent increase of GLO1 activity in the tumor parallel with pair-matched normal tissue.

**Conclusion:** The current findings build up a key-role of enzymatic defense to detoxify cytotoxic AGEs and methylglyoxal levels in tumor cells. These discoveries may give another system to the treatment of breast cancer.

**Keywords:** Breast Neoplasm, Glyoxalase I, Methylglyoxal, Fructosamine-3-kinase, Advanced Glycation End products, Glycation.
